# Correction to “Cardiopulmonary exercise testing and the 2022 definition of pulmonary hypertension”

**DOI:** 10.1002/pul2.12417

**Published:** 2024-07-17

Habedank D, Ittermann T, Kaczmarek S, Stubbe B, Heine A, Obst A, Ewert R. Cardiopulmonary exercise testing and the 2022 definition of pulmonary hypertension. Pulm Circ. 2024 Jun 17;14(2):e12398.

In the abstract, the comparison symbol in the very last term “VE/VCO_2_@AT ≤ 30” is incorrect. This should have read “VE/VCO_2_@AT ≥ 30”.

We apologize for this error.

